# Supplementary material for: The complete genome of 2,6-dichlorobenzamide (BAM) degrader Aminobacter sp. MSH1 suggests a polyploid chromosome, phylogenetic reassignment, and functions of plasmids
Source: Sci Rep. 2021 Sep 23;11:18943. doi: 10.1038/s41598-021-98184-5 (PMC8460812; doi:10.1038/s41598-021-98184-5)
Supplement: Supplementary file 1 — Supplementary Information. [file 41598_2021_98184_MOESM1_ESM.pdf]

Supplementary Information

The complete genome of 2,6-dichlorobenzamide (BAM) degrader *Aminobacter* sp. MSH1 suggests a polyploid chromosome, phylogenetic reassignment, and functions of plasmids.

Running title: Complete genome of *Aminobacter* sp. MSH1

Authors

Tue Kjærgaard Nielsen[a†], Benjamin Horemans[b,c†], Cedric Lood[d,e], Jeroen T'Syen[b], Vera van Noort[d], Rob Lavigne[e], Lea Ellegaard-Jensen[f], Ole Hylling[f], Jens Aamand[g], Dirk Springael[b‡*], Lars Hestbjerg Hansen[a‡*]

[†]These authors contributed equally to the work presented and should be both considered as first author.

[‡] DS and LHH initiated the study and supervised the project equally in separate labs

Institutional Affiliations

[a] University of Copenhagen, Faculty of Science, Department of Plant and Environmental Sciences, Section for Microbiology and Biotechnology, Copenhagen, Denmark

[b] KU Leuven, Faculty of Bioscience Engineering, Department of Earth and Environmental Sciences, Division of Soil and Water Management, Leuven, Belgium

[c] BAT Knowledge Centre, Sustainable Materials Unit, Vlaams Instituut voor Technologisch Onderzoek, Mol, Belgium

[d] KU Leuven, Faculty of Bioscience Engineering, Department of Microbial and Molecular Systems (M²S), Centre of Microbial and Plant Genetics, Leuven, Belgium

[e] KU Leuven, Faculty of Bioscience Engineering, Department of Biosystems, Laboratory of Gene Technology, Leuven, Belgium

[f] Aarhus University, Department of Environmental Science, Section of Environmental Microbiology and Circular Resource Flow, Roskilde, Denmark

[g] Geological Survey of Denmark & Greenland (GEUS), Department of Geochemistry, Copenhagen, Denmark

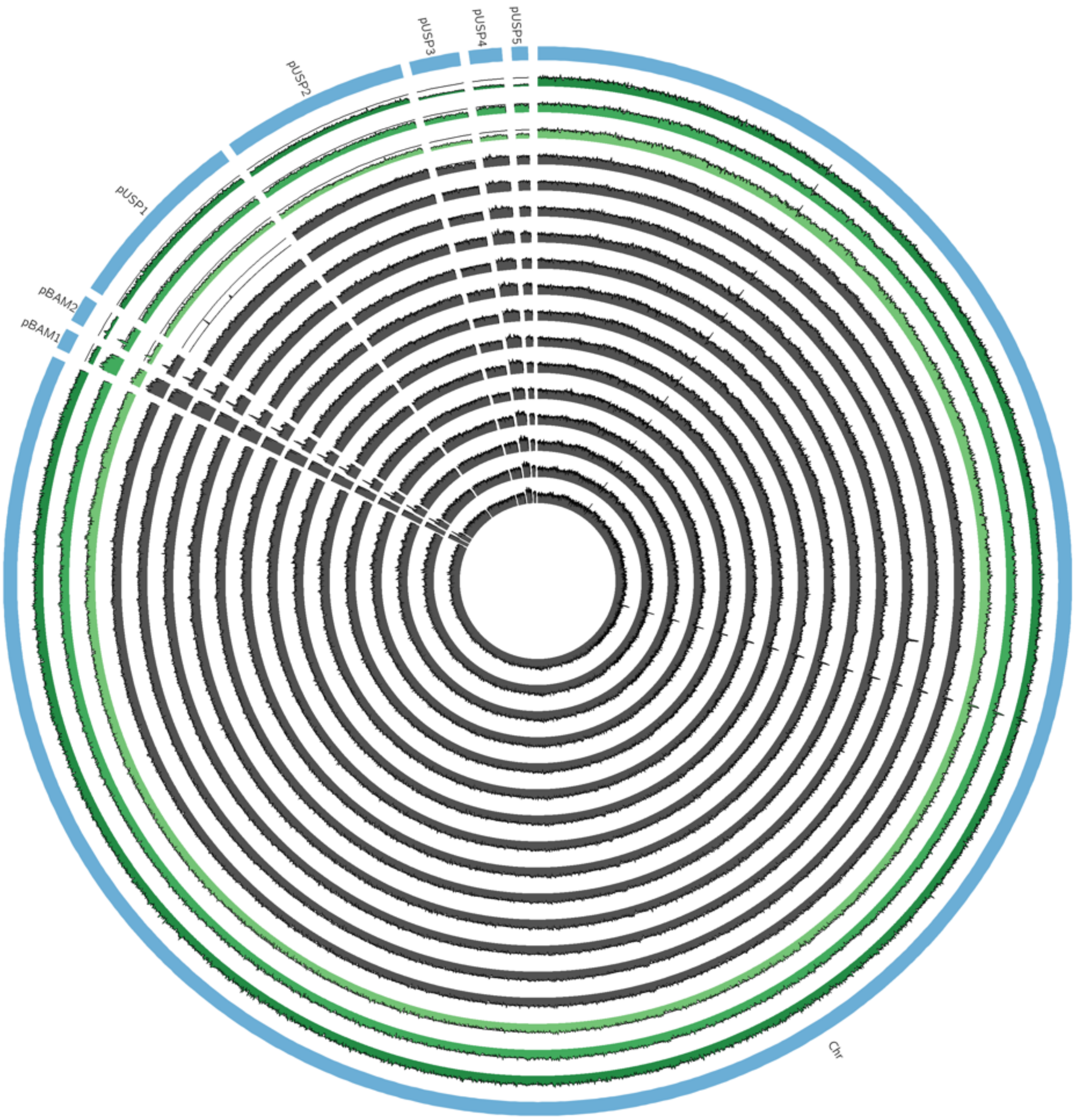

Supplementary Figure S1. Illumina reads mapped to the chromosome and plasmids of MSH1 (DK1 substrain). The outer blue ring indicates the replicons. The inner rings show read mapping coverage of the replicons, normalized to the coverage of the chromosome per replicate, for all of the 17 sequenced replicates. The three first green rings show replicates cryostock, first colony from R2A plate, first colony from R2B broth, respectively. The subsequent 14 grey rings show the replicates that all originate from the same first colony. A solid line in the background of all tracks indicate the chromosome coverage line. Coverage above this line indicates a replicon copy number higher than 1 per chromosome and vice versa.

Supplementary Table S1. Digital DNA:DNA hybridization (dDDH) results from the online Type Strain Genome Server (TYGS) analysis. d0, d4, d6 refers to different algorithms used TYGS. Formula d0 (a.k.a. GGDC formula 1): length of all HSPs divided by total genome length. Formula d4 (a.k.a. GGDC formula 2): sum of all identities found in HSPs divided by overall HSP length. Formula d6 (a.k.a. GGDC formula 3): sum of all identities found in HSPs divided by total genome length. C.I.: Confidence interval.

Type Strain Genome Server								
**Query strain**	**Subject strain**	**dDDH (d0, in %)**	**C.I. (d0, in %)**	**dDDH (d4, in %)**	**C.I. (d4, in %)**	**dDDH (d6, in %)**	**C.I. (d6, in %)**	**G+C content difference (in %)**
**MSH1**	*Aminobacter niigataensis DSM 7050*	69.7	[65.8 - 73.3]	82.5	[79.7 - 85.0]	74.3	[70.8 - 77.5]	0.51
**MSH1**	*Aminobacter aganoensis DSM 7051*	53.4	[49.9 - 56.9]	40.2	[37.7 - 42.7]	50.5	[47.5 - 53.6]	1.01
**MSH1**	*Aminobacter lissarensis DSM 17454*	40.6	[37.2 - 44.0]	30.8	[28.4 - 33.3]	37.4	[34.5 - 40.5]	0.19
**MSH1**	*Aminobacter aminovorans DSM 7048*	41.3	[37.9 - 44.8]	30	[27.6 - 32.5]	37.7	[34.8 - 40.8]	0.31
**MSH1**	*Aminobacter ciceronei DSM 15910*	39.2	[35.8 - 42.6]	29.8	[27.4 - 32.3]	36	[33.1 - 39.1]	0.18
**MSH1**	*Aminobacter ciceronei DSM 17455*	39.2	[35.8 - 42.6]	29.8	[27.4 - 32.3]	36	[33.1 - 39.1]	0.18
**MSH1**	*Chelatobacter heintzii DSM 10368*	38.7	[35.3 - 42.2]	29.7	[27.3 - 32.2]	35.7	[32.7 - 38.7]	0.26
**MSH1**	*Mesorhizobium plurifarium ORS 1032*	18.9	[15.8 - 22.5]	21.9	[19.6 - 24.3]	18.5	[15.9 - 21.5]	1.19
**MSH1**	*Mesorhizobium waimense ICMP 19557*	19.1	[16.0 - 22.7]	21.7	[19.5 - 24.2]	18.7	[16.0 - 21.7]	0.49
**MSH1**	*Mesorhizobium australicum WSM2073*	19.2	[16.1 - 22.8]	21.7	[19.4 - 24.1]	18.8	[16.1 - 21.8]	0.05
**MSH1**	*Mesorhizobium qingshengii CGMCC 1.12097*	19.5	[16.3 - 23.1]	21.6	[19.3 - 24.0]	19	[16.3 - 22.0]	0.24
**MSH1**	*Mesorhizobium sangaii DSM 100039*	19.5	[16.3 - 23.1]	21.6	[19.4 - 24.1]	18.9	[16.3 - 22.0]	0.51
